# The Pedigree of the Central Incisor

**Published:** 1893-11

**Authors:** A. H. Thompson

**Affiliations:** Topeka, Kan.


### THE PEDIGREE OF THE CENTRAL INCISOR.[1]

BY DR. A. H. THOMPSON, TOPEKA, KAN.

THE condition of the upper central incisor in man is something unique, in that while all the other teeth are reduced in form and degraded in specialization, more or less, the central has not only preserved its special form and maintained the practice of its function, but has indeed advanced somewhat, being rather more highly specialized in man, as an effective cutting-instrument, than in some of the lower forms. Its form in man is similar to that of the apes, but the remainder of the teeth of the human denture have degenerated from the completeness of specialization exhibited in those forms.

The lateral incisor, while highly developed in most individuals, is frequently reduced in form and sometimes is totally absent. This is never exhibited in the central incisor, but it is the case with the cuspid. The bicuspids, molars, and lower incisors also show the effects of degenerative modification; the central incisor alone maintains a high degree of completeness and specialization in man, which fact makes a study of its genetic evolution a matter of peculiar interest.

The function of dividing and cutting food is performed by various organs throughout the animal kingdom, and even teeth for cutting are developed very low in the scale of life. The cephalopods have cutting-teeth placed on the odontophore, but these are not true incisors. The insects and crustaceans cut by means of the mandibles, and the "pinchers" of the anterior locomotive organs, which are not true teeth. The leech, neresis, and other worms have saw-like mandibles which are not properly cutting-teeth. In fact, the lowest form in which teeth with any approach to the true incisor form are found, is the sea-urchin, *Echinus*, which has five incisors arranged around a central point, in the remarkable apparatus called "Aristotle's Lantern." They are used with great force

---

[1] Read before the World's Columbian Dental Congress, Chicago, 1893.

to cut shells and rocks. These teeth are set in true alveoli, and are worked by powerful muscles. This is the lowest form in which true incisors are found.

In the fishes there are no incisors, properly so called, unless we consider as such the cutting-teeth of *Sargus*, etc., for the so-called teeth are ankylosed to the maxillary bones.

None of the reptiles have cutting-teeth proper; all of their teeth are pointed for seizing and holding prey. The beaks of turtles are analogous to incisors, but are not homologous with them. The same may be said of the bills and beaks of birds.

Most of the lower mammals are deficient in regard to incisors, usually having teeth on the sides of the jaws for grinding purposes only. The kangaroo has large cutting incisors, as it is an exclusive vegetable-feeder. Others of the marsupialia are variously armed, as they may be herbivorous, carnivorous, or insectivorous. In the rodents we find the central incisors developed wonderfully into the long, continuously growing implements used for cutting.

In the herbivora all of the incisors and also the canines are highly developed for cutting purposes. A curious exception is noted in the ruminants, the most of which have no incisors in the upper jaw. In the carnivorous animals the canines being excessively developed and the cutting function being usurped by the long-bladed sectorials, the incisors are much diminished. In the elephant and mastodon the central incisors are developed into long tusks, which are employed as effective digging implements and piercing weapons.

With all these, however, man has little relationship. With those that remain to be noticed we find the path of the evolution of man and the stages through which his teeth have passed to reach their present forms.

Beginning with the insectivora, the living representatives of an order which was the apparent predecessor of the quadrumana through the lemuridæ, we observe that this order presents many remarkable forms of the central incisor. In some of the moles the incisors are small, and in others like a canine with deep grooves. Owen says, "In the shrews the central is very large, with a large talon on the basal ridge, making a deep notch into which the procumbent lower central closes with a hook-like point." This talon, with the deep groove or sulcus, is sometimes reproduced in man, with the characteristic backward curve of the body of the tooth. "The typical shrew usually manifests rodent analogy by the superior size of the anterior pair of incisors in both upper and lower jaws." But this resemblance is only in regard to general contour. "In *Sorex* the large upper incisors appear bifurcate, from the great development of the posterior talon." This talon is often repeated on the lateral incisor in man, where a lingual cingulum is not uncommon. "The hedge-hog has large centrals, sometimes with a large interspace." This is perhaps the precursor of the large interspace which is often present in man, and is so frequently a matter of heredity.

In the cheiroptera, which are closely related to the insectivora, the incisors are most variable and may be entirely absent. Some of the curious forms exhibited in the insectivorous series are passed on and reappear in the quadrumana, and occasionally in man.

In approaching the quadrumana, the highest branch of animal life related to man by collateral descent from a common ancestor, the lowest family of the order is that of the lemurs. Some of those are closely related to the insectivora as regards form, habits, and dentition.

The lemurs present considerable variation in the form of the central incisor, which advertise relationships with many groups below them and present forms which are very aberrant. Tomes says, "Most of the lemurs have upper incisors which are small and widely separated from each other." One of the lowest, and one closely related to the insectivora, is the little flying galeopithecus. Owen says, "In the colugo the two anterior incisors of the upper jaw are separated by a wide interspace. In the Philippine colugo these teeth are small with a simple bilobed crown, but in the galeopithecus the crown is expanded into a plate with three or four tubercles. . . . The lower centrals present the form of a comb produced by the deeper extension of the marginal notches into the crown. These are analogous to those on the edges of the human incisor, but the notches are more numerous and deeper." This tendency to division is recalled by the tubercles upon the edges of the incisors of man when first erupted. The longitudinal ridges which lead away from the tubercles are also suggestions of this primitive division of the crown, as an embryonic record of the history of its evolution. This was apparent to the great anatomist Owen before the day of philosophic evolution, and in our day the lesson is beautiful and striking.

In other forms of the lemurs the incisors are projecting, long, and narrow, and interdigitate with the procumbent lower incisors, which pass between them for cutting vegetable fiber.

The lowest monkeys, the platyrrhines (or wide-nosed), the

American species, are closely related to the lemurs in many respects, notably in having the third premolar. The central incisor begins to approach the final form in the higher groups, but is still somewhat aberrant. Like the lemurs, the incisors of both jaws are directed more obliquely forward, and so are less vertical than in the higher apes and in man. As these are approached, the incisors become more erect, and the form of the crown is, in consequence, less curved, for in the higher primates it is straighter and more in a line with the long axis of the root. In the platyrrhines it is still more or less of a scoop shape, resembling the lemurs. The scoop shape is often recalled in man, with the characteristic curve and thin edge.

In the catyrrhines (narrow-nosed), the Old World monkeys, the centrals are much wider and larger than the laterals, but nearer the shape found in man, even in the lower form. In some of the baboons there is more or less of a basal ridge, which is often greatly developed on the lingual face. This lingual ridge is often recalled in man, but not on the labial face.

In all the anthropomorpha the centrals are much larger than the laterals, are more vertical than in the lower forms, and assume the final shape presented in man. There is little real difference in the form of the central incisors between the higher apes and man, except in size and quality of structure and in the comparative size of the centrals and laterals. In man the extreme disproportion manifested in the apes is much reduced,—*i.e.*, the centrals are smaller and the laterals are larger.

The gibbons are the lowest of the tailless apes. Their centrals are large and strong, nearing the final human form, except that they project and are somewhat of a curved form. In the orangs the centrals are of great size, are twice the size of laterals, and have the basal ridge. This extreme size is recalled in man by those examples which we see of excessively large centrals. Tomes says these teeth are similar to those of man, but larger. In the chimpanzee the centrals are very much reduced from the size of the orang, and approach the proportions of the same teeth in man, but with a prominent basal ridge as in the orang.

In the gorilla the centrals are still nearer the final shape as in man. They are of the same form and proportions, but are larger and of coarser structure than in man, as are all of the teeth of the greatest ape. There is little real difference between the central of the higher apes and man, for it assumes the final shape long before man is reached, and must have taken on this form before the differentiation which separated the human branch from the quadrumanous.

As showing the persistence of type and the singularity of the survival of this tooth in such perfection, we find that the central incisor of man has lost nothing in the course of the evolution of the species, and yet it is little elevated above that of the apes. In fact, the general structure of the denture of man is degraded and primitive. The central has lost nothing in the process of the destructive evolution of the species, for while the other teeth have been degraded, it has maintained its own and is really superior to the others in the retention of the perfection of its functional specialization.

